# Long-term rice-crab coculturing leads to changes in soil microbial communities

**DOI:** 10.3389/fmicb.2024.1395544

**Published:** 2025-01-08

**Authors:** Liang Ma, Tiexin Yang, Liqiang Dong, Zhengyan Pan, Yingying Feng, Zhiqiang Li, Fuyu Sun

**Affiliations:** ^1^Liaoning Rice Research Institute, Shenyang, China; ^2^Liaoning Academy of Agricultural Sciences, Shenyang, China

**Keywords:** rice-crab coculture, soil physicochemical properties, soil enzyme activity, soil bacterial community, soil fungal community

## Abstract

**Purpose:**

In order to investigate the effects of a rice-crab coculture mode and its duration on the richness and diversity of the soil microbial community.

**Method:**

Soil from long-term rice-crab coculture mode (MY), newly established rice-crab coculture mode (OY) and rice monoculture mode (N) were used to measured soil physicochemical properties, enzyme activity and 16S and ITS soil microbial communities.

**Results:**

The results revealed that in terms of mode, the MBC, MBN and CAT of OY were significantly greater than those of N by 10.75, 23.47 and 30.71% (*p* < 0.05), respectively. The richness and diversity of the soil microbial communities changed little, and there was no difference in the main species. In terms of duration, the OM, SC and PPO contents of MY were significantly greater than those of OY by 21.96, 41.89 and 11.52% (*p* < 0.05), respectively. The soil fungi changed significantly, and the main species were *Mortierella* and *Pseudeurotium* in genus level. The soil physicochemical properties and soil enzymes explained 93.38 and 93.66%, respectively, of the variation in the soil microbial community, and OM and DHA were the main factors influencing the change in soil biodiversity.

**Conclusion:**

Our results suggested that long-term rice-crab coculture mode altered the richness and diversity of the soil microbial community and soil carbon sequestration.

## 1 Introduction

An agricultural coculture system is a comprehensive agricultural production system that combines agricultural planting and aquaculture (Prein, 2002). Compared with the rice monoculture mode, the coculture mode utilizes the symbiotic and complementary relationships between plants and animals to realize the efficient circulation of matter and energy in the production process, which can effectively reduce the dependence on exogenous fertilizers and pesticides (Cavalett et al., 2006). Moreover, the coculture mode can not only improve economic and ecological benefits, but also positively regulate the temperature and humidity of the environment in the production area, which can obtain higher increase ecosystem service values (Arunrat and Sereenonchai, 2022); therefore, it is considered an environmentally friendly sustainable agricultural development mode and a future development direction for agricultural production (Liu et al., 2022).

Soil microorganisms are important for organic matter degradation, the soil carbon cycle and the soil nitrogen cycle (Morrissey et al., 2023; Wang G. et al., 2020; Paul, 2016), and the richness and diversity of these microorganisms affect the material and energy cycles in paddy fields (Wang Z. et al., 2020). In recent years, changes in soil microorganisms under the coculture mode have been reported. In a rice-fish coculture system, soil nitrogen fixation and aromatic compound degradation were enhanced, and there were significant differences among *Nitrosporae*, *Rokubacteria*, *GAL15* and *Elusimicrobia* (Arunrat et al., 2022). In the rice-shrimp coculture mode, the daily activity of shrimp can increase the total soil organic matter content, change the soil microbial richness, and cause a shift in the dominant bacterial groups toward *Proteobacteria*, *Bacteroidetes* and *Chloroforma* (Yang et al., 2022). In a rice-crab coculture system, the input of crab feed increased the protein content in soil and significantly reduced the diversity of saprophytic *macrofungi* (Li et al., 2021). Differences in environmental factors are thought to cause changes in the microbial communities among different systems.

In agricultural systems, several studies on the influence of environmental factors on microbial communities have been conducted. Small soil aggregates (<0.5 mm) and large soil aggregates (≥0.5 mm) have more OTUs (Sun et al., 2021). Under drought stress, plant roots can secrete fatty acids and diterpenoid metabolites, which have been shown to directly affect the *α* and *β* diversities of soil microorganisms (Bi et al., 2021). Soil water and the carbon/nitrogen ratio are also important factors affecting the composition of soil microbial communities and can explain more than 90% of the spatial variation in soil microbial communities (Wu et al., 2020). However, because of the strong buffering that occurs in soils in the coculture mode, changes in the α diversity of soil microorganisms cannot take place in a short time (Arunrat et al., 2022). Currently, alterations within the soil microbial community under the coculture mode remain confined to a preliminary, short-term phase, whereas comprehensive scientific data pertaining to the long-term effects of this coculture mode on the soil microbial community are still conspicuously absent (Wang et al., 2024; Ren et al., 2023; Li et al., 2022). Therefore, the investigations of microbial richness and diversity in the coculture mode should be ongoing.

In this study, the duration of the rice-crab coculture mode was accounted for when considering the changes in the soil microbial communities in the long-term coculture mode. We hope to address (1) the changes in soil microbial community richness and diversity in a long-term coculture mode and (2) the relationships among soil properties, enzyme activity and microbial communities. We compared the characteristics of the soil bacterial and fungal communities among the conventional rice monoculture mode, newly established rice-crab coculture mode and long-term rice-crab coculture mode and analyzed the correlations between the soil physicochemical properties and enzyme activity, between the soil physicochemical properties and microbial communities and between the enzyme activity and microbial communities. The results are also helpful for understanding and predicting the variation characteristics of soil microbial community richness and diversity in the coculture mode and provide information for exploring the matter and energy cycle in this mode.

## 2 Materials and methods

### 2.1 Study site

Our experimental site is located in the city of Panjin, Liaoning Province (41°19′34″ N; 122°3′52″ E), in the alluvial plain of the lower reaches of the Liaohe River and belongs to the single-cropping rice region in northern China. The soil is slightly alkaline or alkaline, and the pH (water) is between 8.0 and 8.8. The experimental site is located in Raoyang village, an area that was used for conventional rice cultivation before 2000. The rice-crab coculture mode was introduced in 2000, and the coculture paddy field area has increased annually since then.

### 2.2 Experimental design

To investigate the long-term effects of the rice-crab coculture mode on soil microbial community succession, 3 adjacent paddy fields were selected in 2022: a long-term rice-crab coculture mode field (20 years, MY), a newly established rice-crab coculture mode field (1 year, OY) and a conventional rice monoculture mode field (N). N is a paddy field that has been planted with rice for more than 40 years and has never raised crab or other aquatic animals artificially. Each selected paddy field was greater than 1 hm^2^ in area. The stocking amount of the juvenile crab in the paddy field was 75 kg/hm^2^ 3–5 days before transplanting, and the weight of each crab was approximately 7 g. The crabs were fed special feed, which was 3–5% of the weight of the crabs. Afterward, according to the consumption of crab feed on the previous day, the feeding amount of crab feed consumed increased or decreased appropriately (Dong et al., 2023). The rice variety used was Yanjing 935 (*Oryza sativa, japonica*). The rice seedlings were transplanted in mid-May, and the mitten crab seedlings were released the following week. Before rice seedling transplantation, 525 kg/hm^2^ of compound fertilizer (N: P_2_O_5_: K_2_O = 27:13:15) was applied to the MY and OY treatments; the subsequent fertilizer came from the residual feed that the crab had not ingested and from the dung of the crab. In the N treatment, the same compound fertilizer was applied to the rice seedlings at a rate of 1,050 kg/hm^2^ before transplanting, and diammonium hydrogen phosphate (150 kg/hm^2^) was added as spike fertilizer at the rice heading stage. Herbicides and insecticides were not used during the rice growing period of in MY and OY, whereas herbicides and insecticides were applied as needed at various stages of rice growth in N.

### 2.3 Sample collection

On July 14, 2022, soil samples (0–20 cm) were collected from the three experimental fields. Three sampling areas were randomly selected for each treatment, and the area sampled was 10 m × 10 m. In each selected area, soil samples were collected via a 5-point sampling method. From each area, five soil samples were randomly collected from 0 to 20 cm soil layer, after which the roots and straw residues were removed. The fresh soil samples were mixed and divided into three parts. The first part was stored at −80°C for analysis of the soil microbial community. The second part was stored at 4°C in a refrigerator for the analysis of soil enzyme activity within 7 days (Che et al., 2023; Tang et al., 2021). The third part was dried and ground with a 2 mm sieve for the analysis of soil physicochemical properties.

### 2.4 Measurements and analyses

The soil microbial communities were analyzed by sequencing the V3-V4 and ITSF regions via high-throughput sequencing, which was performed by the Allwegene Company. For the V3-V4 region, the initial primer 338F (ACTCCTACGGGA-GGCAGCAG) and posterior primer 806R (GGACTACHVGGGTWTCTAAT) were used to sequence the V3-V4 region, and the amplified fragment length was 468 bp. The initial ITS1-F primer used was CTTGGTCATTTAGAGGAAGTAA, the ITS2 posterior primer was TGCGTTCTTCATCGATGC, and the amplified fragment length was 425 bp.

The soil enzyme activity was measured via kits according to the manufacturer’s recommendations, as shown in Table 1.

**Table 1:** Soil enzyme activity test details.

Index	Assay kit	Manufacturer
Urease	Soil Uricase (S-UR) Activity Assay Kit	Boxbio, China
Protease	Soil Protease (S-NPT) Activity Assay Kit	Boxbio, China
Dehydrogenase	Soil Dehydrogenase (S-DHA) Activity Assay Kit	Boxbio, China
Catalase	Soil Catalase (S-CAT) Activity Assay Kit	Boxbio, China
Acid Phosphatase	Soil Acid Phosphatase (S-ACP) Activity Assay Kit	Boxbio, China
Neutral Phosphatase	Soil Neutral Phosphatase (S-NP) Activity Assay Kit	Boxbio, China
Alkaline Phosphatase	Soil Alkaline Phosphatase (S-AKP/ALP) Activity Assay Kit	Boxbio, China
Sucrase	Soil Sucrase (S-SC) Activity Assay Kit	Boxbio, China
Polyphenol Oxidase	Soil Polyphenol Oxidase (S-PPO) Activity Assay Kit	Boxbio, China
Cellulase	Soil Cellulase (S-CL) Activity Assay Kit	Boxbio, China
β-Xylosidase	Soil β-Xylosidase (S-β-XYS) Activity Assay Kit	Boxbio, China

The soil physicochemical properties were determined according to previous studies (Che et al., 2023; Xu et al., 2022; Li et al., 2022; Liu et al., 2018). The soil pH was determined via a pH meter in soil-to-water (W/V, 1:2.5) mixtures of dry soil and distilled water; the total nitrogen content (TN) was measured via Kjeldahl digestion and distillation azotometry; the available nitrogen content (AN) was measured via the microdiffusion method after alkaline hydrolysis; the total phosphorus content (TP) was measured via the molten NaOH method and an ultraviolet spectrophotometer; the available phosphorus content (AP) was measured via an ultraviolet spectrophotometer following NaHCO_3_ extraction; the total potassium content (TK) was measured via the molten NaOH method and an atomic absorption spectrophotometer; the available potassium content (AK) was determined via an atomic absorption spectrophotometry after neutral extraction with CH_3_COONH_4_; the soil organic matter content (OM) was measured via FeSO_4_ titration after wet oxidation with K_2_Cr_2_O_7_-H_2_SO_4_ external heating; the cation exchange capacity (CEC) was determined via the EDTA-CH_3_COONH_4_ exchange method; and the microbial biomass carbon (MBC) and the microbial biomass nitrogen (MBN) were determined via chloroform fumigation extraction.

### 2.5 Data statistics and analysis

Least significant difference (LSD) analysis of variance was used to analyze the soil physicochemical properties of the N, OY and MY treatments. One-way analysis of variance (ANOVA) with Duncan’s multiple comparisons test was used to analyze differences between different treatments. Test results with *p* < 0.05 and *p* < 0.01 indicate a significant difference between treatments and a very significant difference. Redundancy analysis (RDA) was performed using Canoco software (version 5.0) to determine the correlations between the soil microbial communities and soil physicochemical properties. The data in the tables and graphs are presented as the means ± standard errors. Microsoft Excel 2019 (Microsoft, USA) was used for data collation, SPSS 22.0 (IBM, USA) was used for data analysis, and Origin 2021 (Origin Lab, USA) was used for plotting.

## 3 Results and analysis

### 3.1 Differences in soil physicochemical properties

The results of the soil physiochemical analyses showed differences between the conventional rice monoculture mode and the rice-crab coculture mode, and the duration of the rice-crab coculture mode also affected the soil physicochemical properties (Table 2). Compared with those of the N treatment, the TN, TP, AN, AP and AK contents of the OY treatment significantly decreased by 23.21, 22.81, 16.67, 36.77 and 23.08% (*p* < 0.05), respectively; the MBC and MBN contents of the OY treatment significantly increased by 10.75 and 23.47%, respectively (*p* < 0.05). These results indicated that the coculture mode could change the soil physicochemical properties. Compared with those of the OY treatment, the soil pH and TK in the MY treatment were significantly decreased by 1.76 and 5.41%, respectively (*p* < 0.05), and the OM in the MY treatment significantly increased by 21.96% (*p* < 0.05). These results indicated that the duration of the coculture mode could also change the soil physicochemical properties.

**Table 2:** Soil physicochemical properties in different treatments (mean + SE, *n* = 3).

	pH	TN	TP	TK	AN	AP	AK	OM	CEC	MBC	MBN
MY	8.39 ± 0.03b	0.88 ± 0.02b	0.44 ± 0.02b	21.85 ± 0.33b	57.85 ± 0.62b	9.16 ± 0.17b	280.3 ± 11.3b	21.06 ± 0.51a	17.21 ± 0.64a	105.73 ± 1.13a	9.33 ± 0.35a
OY	8.54 ± 0.03a	0.86 ± 0.02b	0.44 ± 0.01b	23.10 ± 0.23a	57.85 ± 1.25b	12.71 ± 1.79b	251.3 ± 8.1b	17.27 ± 0.47b	17.28 ± 0.38a	101.32 ± 0.74a	8.47 ± 0.28a
N	8.57 ± 0.04a	1.12 ± 0.03a	0.57 ± 0.01a	23.06 ± 0.34a	69.42 ± 2.16a	20.10 ± 1.19a	326.7 ± 11.7a	17.40 ± 0.64b	17.07 ± 0.29a	91.48 ± 2.56b	6.86 ± 0.10b

TN is total nitrogen (g/kg); TP is total phosphorus (g/kg); TK is total potassium (g/kg); AN is available nitrogen (mg/kg); AP is available phosphorus (mg/kg); AK is available potassium (mg/kg); OM is organic matter (g/kg); CEC is cation exchange capacity (cmol/kg); MBC is microbial biomass carbon (mg/kg); MBN is microbial biomass nitrogen (mg/kg). Different letters in the same column indicate significant differences at the *p* < 0.05 level.

### 3.2 Differences in soil enzyme activity

Changes in soil enzyme activity are closely related to soil physicochemical properties and can also reflect the richness and diversity of the soil microbial community. The analysis of the soil enzyme activity among the different treatments showed that the rice-crab coculture mode led to the regulation of the activity of some soil enzymes (Table 3). NPT, ACP, NP and *β*-XYS were stable and there was no significant difference among the treatments. Compared with those of the N treatment, the UR, ALP, SC and PPO of the OY treatment significantly decreased by 28.57, 29.46, 25.85 and 12.12%, respectively (*p* < 0.05); the CAT activity of the OY treatment was significantly increased by 30.71% (*p* < 0.05). These results indicated that the coculture mode could change soil the enzyme activity. Compared with those of the OY treatment, the DHA, CAT and CL of MY significantly decreased by 70.45, 32.95, and 32.62%, respectively (*p* < 0.05); the SC and PPO contents of the MY treatment were significantly increased by 41.89 and 11.52%, respectively (*p* < 0.05). These results indicated that duration of coculture mode could change soil enzyme activity.

**Table 3:** Soil enzyme activity in different treatments (mean ± SE, *n* = 3).

	UR	NPT	DHA	CAT	ACP	NP	ALP	SC	PPO	CL	β-XYS
MY	0.068 ± 0.003b	9.35 ± 0.22a	4.64 ± 0.76b	2.34 ± 0.13b	2.87 ± 0.03a	0.22 ± 0.01a	0.88 ± 0.07b	20.39 ± 0.55a	0.67 ± 0.01a	5.64 ± 0.16b	92.13 ± 3.31a
OY	0.070 ± 0.001b	10.68 ± 0.45a	15.70 ± 2.86a	3.49 ± 0.10a	2.85 ± 0.02a	0.21 ± 0.01a	0.79 ± 0.03b	14.37 ± 1.31b	0.58 ± 0.01b	8.37 ± 0.33a	91.51 ± 2.00a
N	0.098 ± 0.006a	10.47 ± 0.44a	13.45 ± 0.81a	2.67 ± 0.03b	2.86 ± 0.05a	0.21 ± 0.01a	1.12 ± 0.01a	19.38 ± 0.90a	0.66 ± 0.02a	9.32 ± 0.80a	92.48 ± 1.44a

UR is soil urease (mg/d/g); NPT is soil protease (μg/g/2 h 50°C); DHA is soil dehydrogenase (μg/g/d); CAT is soil catalase (μmol/min); ACP is soil acid phosphatase (mg/g/d); NP is soil neutral phosphatase (mg/g/d); ALP is soil alkaline (mg/g/d); SC is soil sucrase (mg/d/g); PPO is soil polyphenol oxidase (mg/g/2 h); CL is soil cellulase (mg/d/g); β-XYS is soil β-xylosidase (μg/g/h). Different letters in the same column indicate significant differences at the *p* < 0.05 level.

### 3.3 Differences in soil bacterial community structure and diversity analysis

Alpha diversity, also known as in-habitat diversity, is used to reflect the richness and diversity of microbial communities. The results of the determination of the soil bacterial alpha diversity in the different treatments showed that there was no significant difference among the treatments, while the soil bacterial richness and community diversity of the N treatment were greater than those of the MY treatment were (Table 4). Compared with those of the MY treatment, the Chao 1 index, observed_species index, PD whole-tree index and Shannon index of the N treatment increased by 7.19, 9.62, 7.45 and 4.01%, respectively. Compared with those of the MY treatment, the richness of the soil bacterial community in the OY treatment decreased, but the diversity increased; the Chao 1 index was 5.04% lower; the observed_species index, PD whole tree index and Shannon index were 3.66, 3.13 and 6.18% greater, respectively.

**Table 4:** Alpha diversity of soil bacteria in different treatments (mean ± SE, *n* = 3).

Treatment	Chao 1	Observed_species	PD whole tree	Shannon’s index
MY	5290.06 ± 530.63a	4046.50 ± 416.31a	309.82 ± 26.90a	9.22 ± 0.49a
OY	5023.62 ± 646.76a	4194.57 ± 369.63a	319.53 ± 28.39a	9.79 ± 0.16a
N	5670.44 ± 123.37a	4435.60 ± 109.36a	332.90 ± 8.73a	9.59 ± 0.12a

The same letters within a column indicate no significant difference at the *p* < 0.05 level.

**Figure 1:**
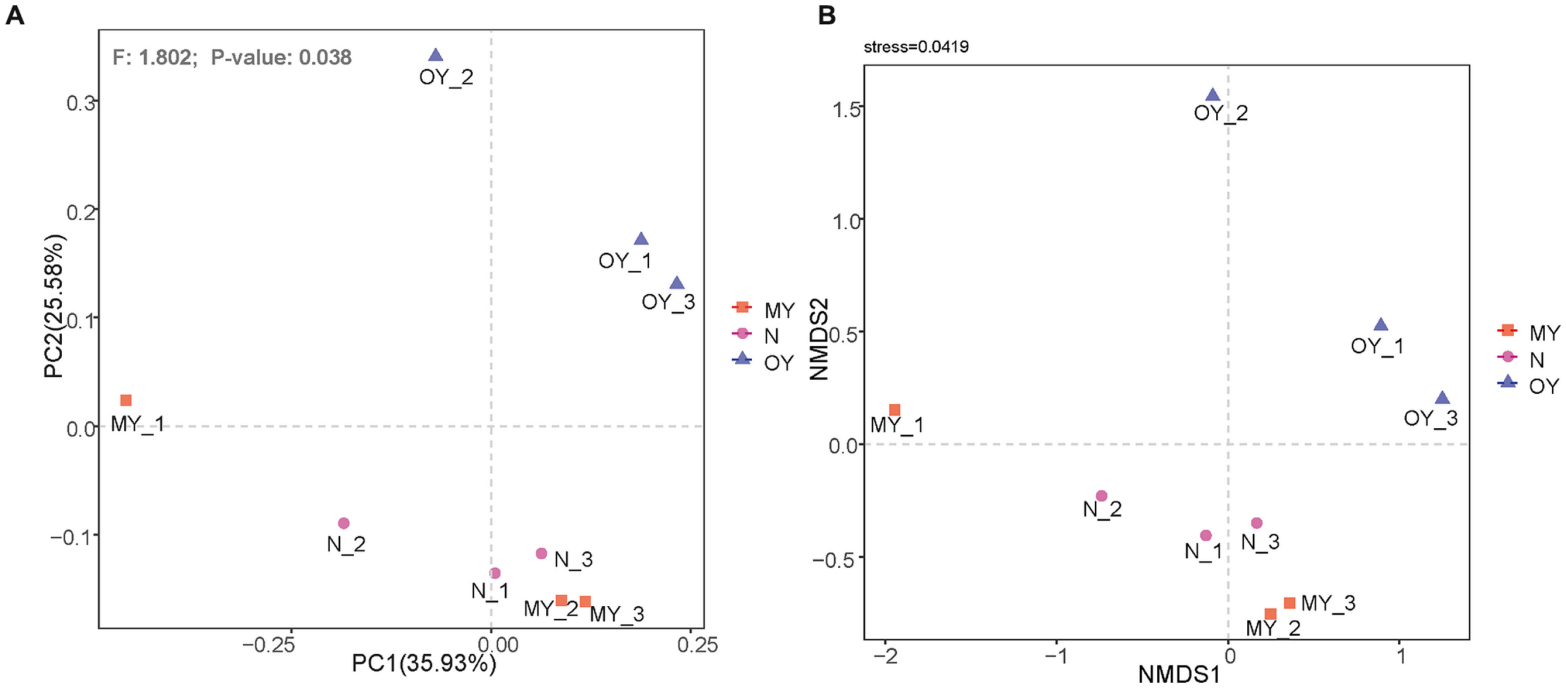
**(A)** Principal coordinates analysis of soil bacterial communities in different treatments. MY indicate a long-term rice-crab coculture mode field, OY indicate a newly established rice-crab coculture mode field, N indicate a conventional rice monoculture mode field. **(B)** Nonmetric multidimensional scaling of soil bacterial communities in different treatments. MY indicate a long-term rice-crab coculture mode field, OY indicate a newly established rice-crab coculture mode field, N indicate a conventional rice monoculture mode field.

Beta diversity, also known as between-habitat diversity, reflects the diversity between different samples or groups. Principal coordinate analysis was used to explore the similarities or differences in the compositions of the soil bacterial communities under the different treatments. The results showed (Figure 1A) that PC1 explained only 35.97% of the differences in bacterial communities under the different treatments, whereas PC2 explained 25.67% of the differences in bacterial communities. These findings indicated other factors affected soil microorganisms. The results of the permutational multivariate analysis of variance indicated that the species composition and relative richness of MY, OY and N significantly differed (*p* < 0.05). Nonmetric multidimensional scaling (nMDS) showed (Figure 1B) that the regression of the results of repetitions of the same treatment was weak but could reflect the real distribution of the test results (stress <0.05).

**Figure 2:**
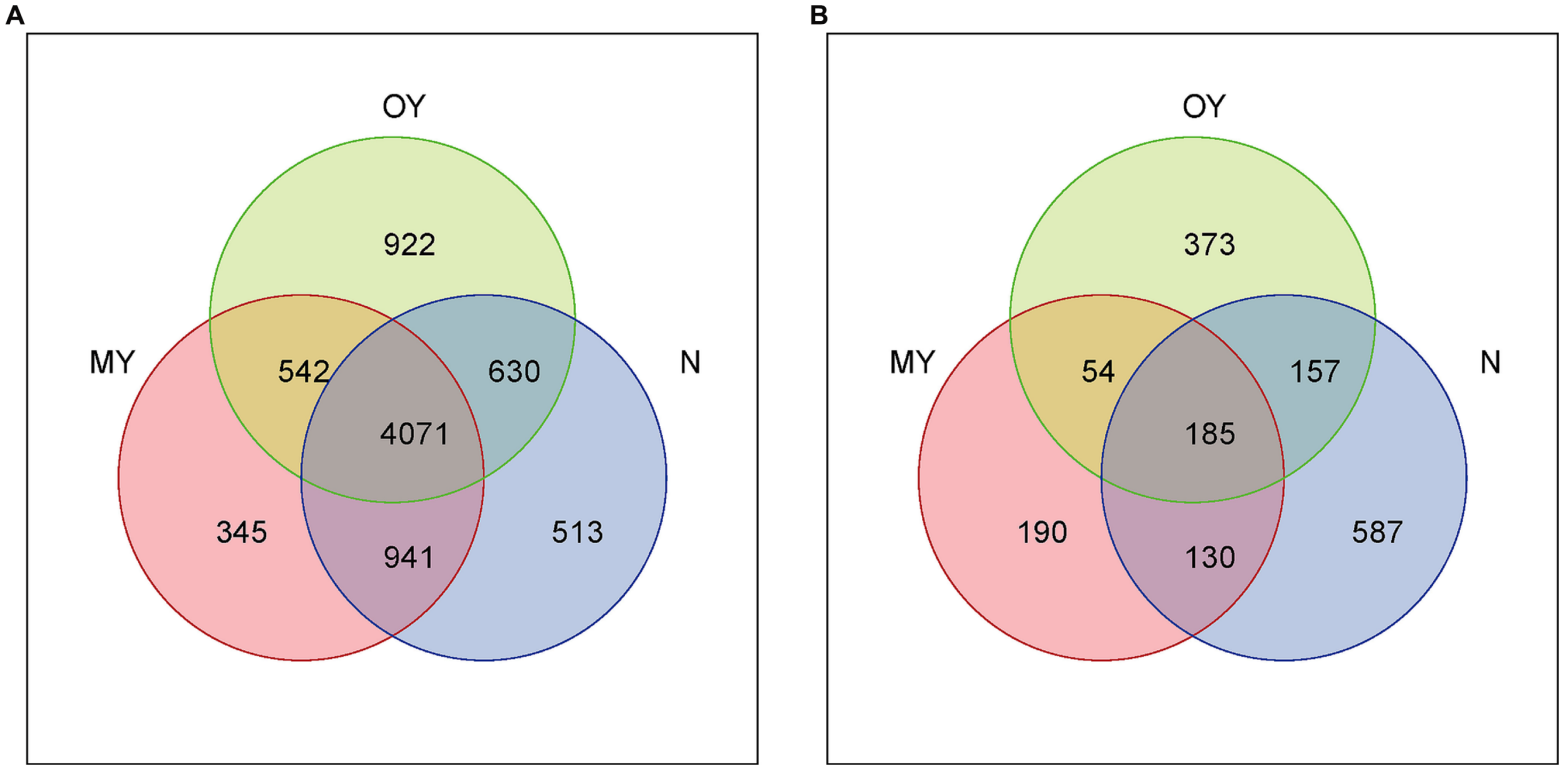
**(A)** Venn diagram of soil bacteria in different treatments based on the distribution of operational taxonomic units (OTUs). MY indicate a long-term rice-crab coculture mode field, OY indicate a newly established rice-crab coculture mode field, N indicate a conventional rice monoculture mode field. **(B)** Venn diagram of soil fungi in different treatments based on the distribution of operational taxonomic units (OTUs). MY indicate a long-term rice-crab coculture mode field, OY indicate a newly established rice-crab coculture mode field, N indicate a conventional rice monoculture mode field.

**Figure 3:**
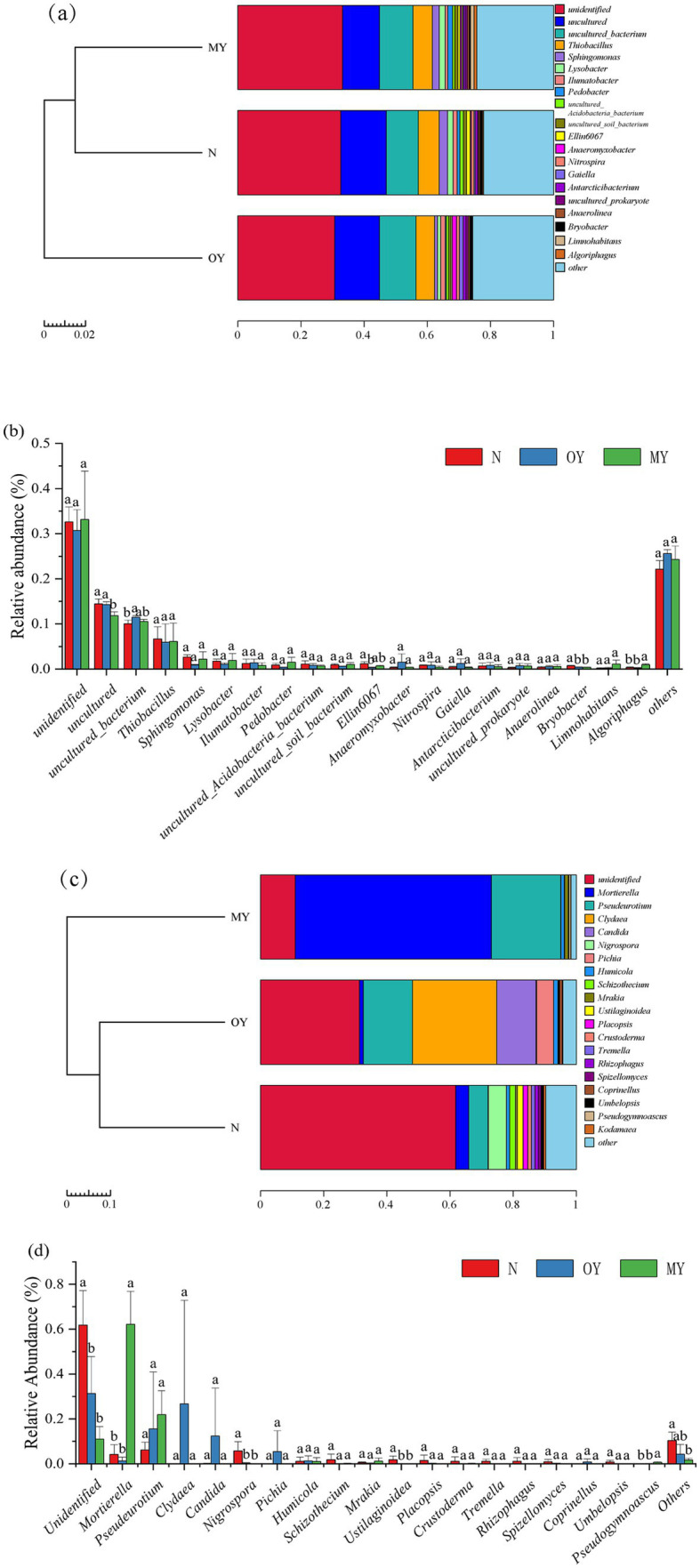
Relative abundances of species at the genus level in different treatments. **(A,B)** Were relative abundance of bacteria in the genus level; **(C,D)** were relative abundance of fungus; MY indicate a long-term rice-crab coculture mode field, OY indicate a newly established rice-crab coculture mode field, N indicate a conventional rice monoculture mode field.

The distributions and compositions of the soil bacterial communities showed differences in the soil bacterial community composition among the different treatments (Figures 2A, 3A,B). There were 345, 522, and 513 unique OTUs in the MY, OY, and N treatments, respectively, and the number of identical OTUs common to all the treatments was 4,071. A total of 682 species of bacteria were detected at the genus level. The results of cluster analysis showed that the soil bacterial community of OY treatment was different from those of N and MY treatments, indicating that the rice-crab coculture mode had a significant effect on soil bacterial community in the short term. Among them, the dominant bacterial groups in the MY, OY and N treatments were *unidentified* (30.77 –33.11%), *uncultured* (11.83 –14.47%) and *uncultured_bacterium* (10.04 –11.46%), and the monomer content was greater than 10%.

### 3.4 Differences in the soil fungal community structure and diversity analysis

The results of the determination of soil fungal alpha diversity in the different treatments showed that the soil fungal richness and community diversity in the N treatment were significantly different from those in the MY treatment (Table 5). Compared with those of the MY treatment, the Chao 1 index, observed_species index, PD whole tree index and Shannon’s index of the N treatment were 59.95, 76.41, 59.95, and 147.43% greater, respectively, and there was a significant difference in the Shannon index. The soil fungal richness and community diversity in the OY treatment were greater than those in the MY treatment, and the Chao 1, observed_species index, PD whole tree index and Shannon’s index were 15.97, 14.45, 49.38, and 16.24% greater, respectively.

**Table 5:** Alpha diversity of soil fungi in the different treatments (mean ± SE, *n* = 3).

Treatment	Chao 1	Observed_species	PD whole tree	Shannon’s index
MY	321.71 ± 120.66a	263.00 ± 84.88a	87.71 ± 17.66a	2.34 ± 0.63b
OY	373.10 ± 305.48a	301.00 ± 283.29a	131.02 ± 11.81a	2.72 ± 1.45b
N	514.57 ± 158.73a	463.97 ± 133.27a	140.29 ± 33.63a	5.79 ± 0.02a

Different letters within a column indicate significant differences at the *p* < 0.05 level.

**Figure 4:**
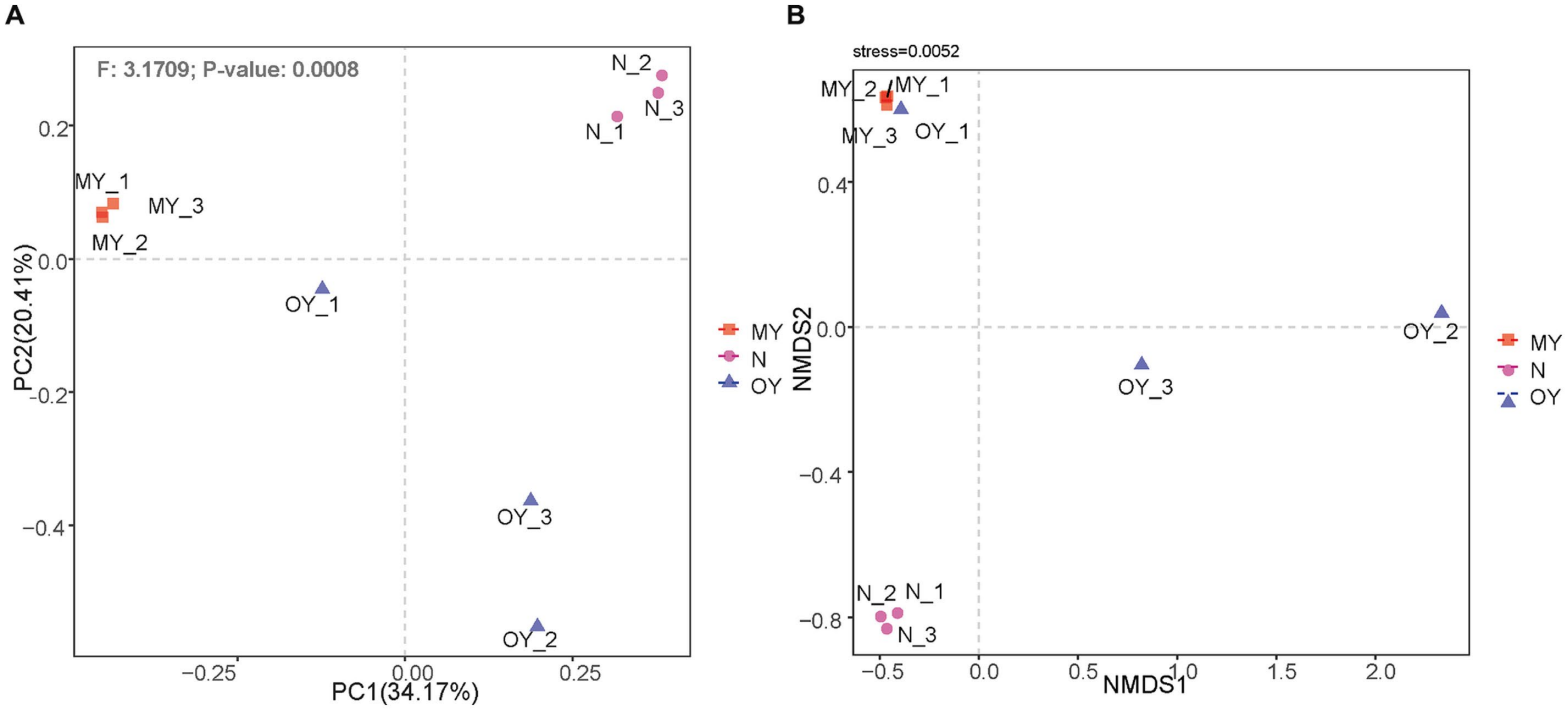
**(A)** Principal coordinates analysis of the soil fungal communities in different treatments. MY indicate a long-term rice-crab coculture mode field, OY indicate a newly established rice-crab coculture mode field, N indicate a conventional rice monoculture mode field. **(B)** Nonmetric multidimensional scaling of the soil fungal communities in different treatments. MY indicate a long-term rice-crab coculture mode field, OY indicate a newly established rice-crab coculture mode field, N indicate a conventional rice monoculture mode field.

Principal coordinate analysis was used to explore the similarities and differences in the compositions of the soil fungal communities under the different treatments. PC1 explained only 34.29% of the differences in fungal communities under the different treatments, and PC2 explained 20.38% of the differences in the fungal communities. The results of PERMANOVA indicated that the species composition and relative richness of MY, OY and N significantly differed (*p* < 0.05) (Figure 4A). The nMDS results showed (Figure 4B) that the regressions of the results of Y20 and N results were strong, whereas the regression of the OY results was weak. Nevertheless, it reflected the distribution of the test results (stress <0.05).

The distribution and composition of the soil fungal communities showed differences in the soil fungal community composition among the different treatments (Figures 2B, 3C,D). The numbers of unique OTUs in the MY, OY, and N treatments were 190, 373, and 587, respectively, and 185 identical OTUs were shared among the treatments. A total of 283 species of fungi were detected at the genus level. The results of cluster analysis showed that the fungi community of MY treatment was different from those of N and OY treatments, indicating that long-term rice-crab cuculture mode had great influence on soil fungal community. The dominant species in the MY treatment were *Unidentified* (10.99%), *Mortierella* (62.15%) and *Pseudeurotium* (21.92%), accounting for more than 80% of all the species. The dominant species in the OY treatment were *Unidentified* (31.32%), *Pseudeurotium* (15.56%), *Clydaea* (26.69%), *Candida* (12.41%), and *Pichia* (5.42%), accounting for more than 80% of all species. The abundance of fungi in the N treatment was relatively *Unidentified* (61.76%), *Mortierella* (4.17%), *Pseudeurotium* (6.11%), and *Nigrospora* (5.69%), accounting for more than 75% of all species.

### 3.5 Correlations between soil physicochemical properties and enzyme activity

**Figure 5:**
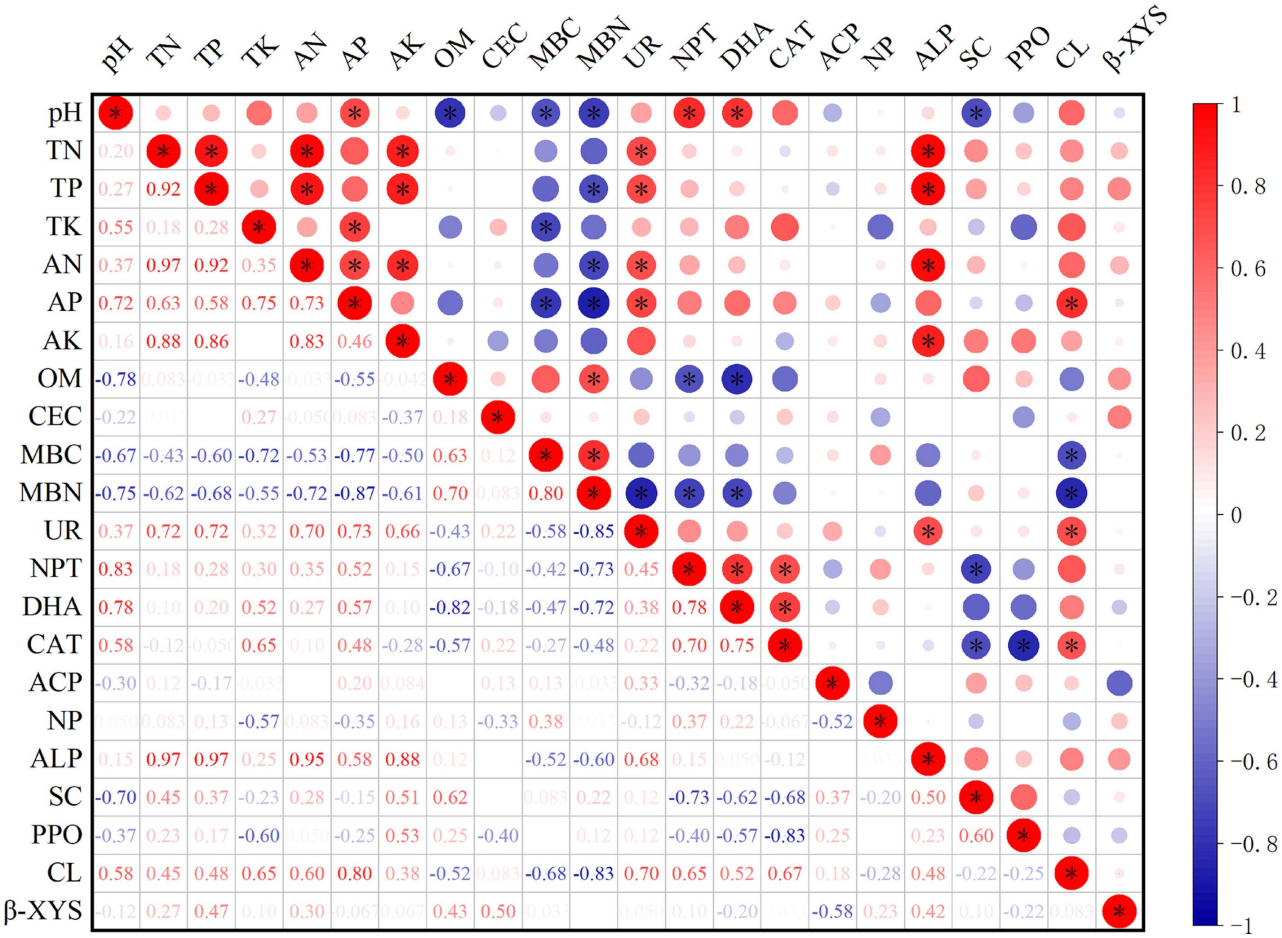
Correlation coefficients between soil physicochemical properties and soil enzymes. TN is total nitrogen (g/kg); TP is total phosphorus (g/kg); TK is total potassium (g/kg); AN is available nitrogen (mg/kg); AP is available phosphorus (mg/kg); AK is available potassium (mg/kg); OM is organic matter (g/kg); CEC is cation exchange capacity (cmol/kg); MBC is microbial biomass carbon (mg/kg); MBN is microbial biomass nitrogen (mg/kg); UR is soil urease (mg/d/g); NPT is soil protease (μg/g/2 h 50°C); DHA is soil dehydrogenase (μg/g/d); CAT is soil catalase (μmol/min); ACP is soil acid phosphatase (mg/g/d); NP is soil neutral phosphatase (mg/g/d); ALP is soil alkaline (mg/g/d); SC is soil sucrase (mg/d/g); PPO is soil polyphenol oxidase (mg/g/2 h); CL is soil cellulase (mg/d/g); and *β*-XYS is soil β-xylosidase (μg/g/h).

Spearman correlation was used to measure the correlation between soil physicochemical properties and enzyme activity (Figure 5). There was a positive correlation and negative correlation between soil physicochemical properties and soil enzyme activity. There was a correlation between soil nutrients but no correlation between OM and CEC. UR was significantly positively correlated with TN, TP, AN, and AP (*p* < 0.05); NPT and DHA were significantly positively correlated with pH (*p* < 0.05); ALP was significantly positively correlated with TP, TN, AN, and AK (*p* < 0.05); and CL was significantly positively correlated with TP (*p* < 0.05). These findings indicated that changes in soil nutrients and pH caused changes in soil enzyme activity.

### 3.6 Factors influencing bacterial and fungal community structure

**Figure 6:**
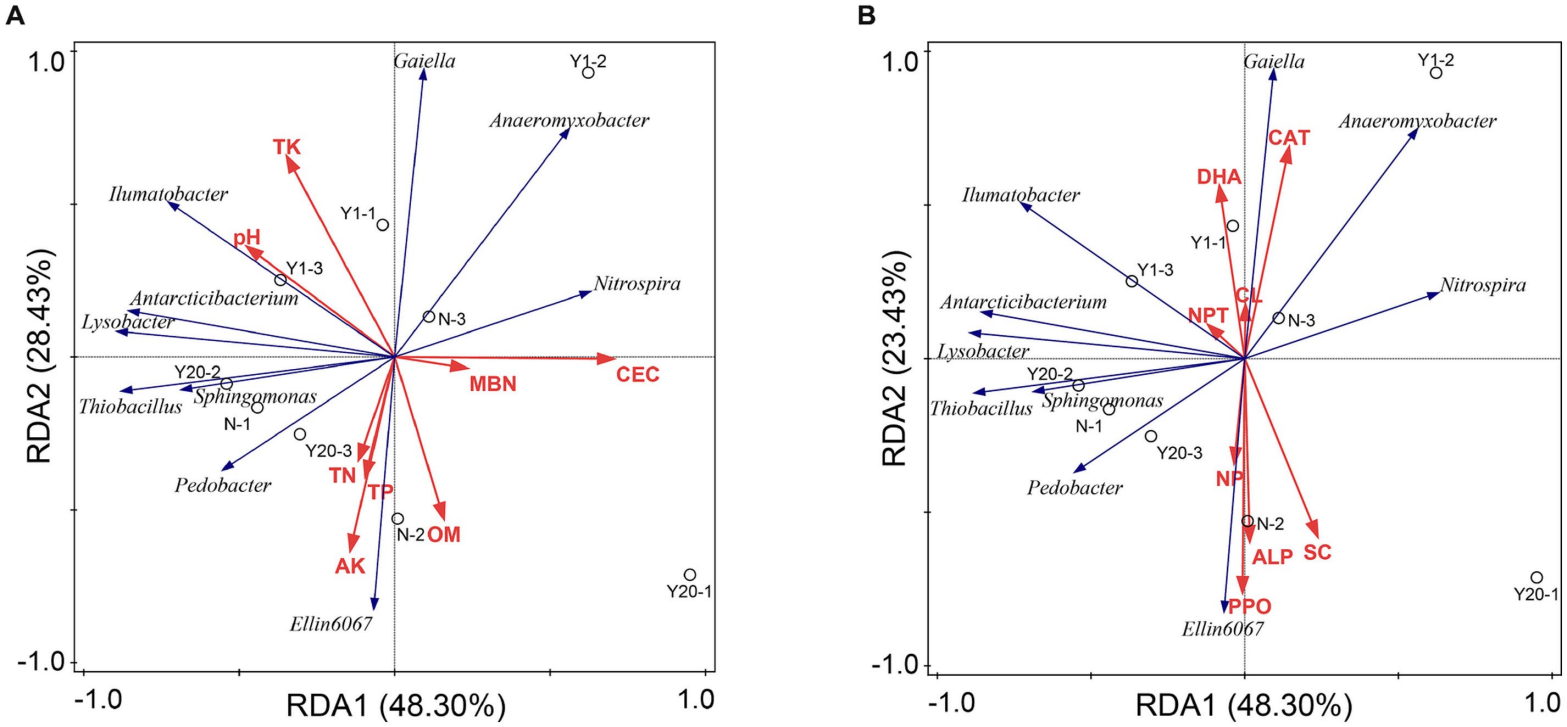
**(A)** Redundancy analysis of bacterial communities and soil properties. TN is total nitrogen (g/kg); TP is total phosphorus (g/kg); TK is total potassium (g/kg); AN is available nitrogen (mg/kg); AP is available phosphorus (mg/kg); AK is available potassium (mg/kg); OM is organic matter (g/kg); CEC is cation exchange capacity (cmol/kg); MBC is microbial biomass carbon (mg/kg); MBN is microbial biomass nitrogen (mg/kg). **(B)** Redundancy analysis of bacterial communities and soil enzymes. UR is soil urease (mg/d/g); NPT is soil protease (μg/g/2 h 50°C); DHA is soil dehydrogenase (μg/g/d); CAT is soil catalase (μmol/min); ACP is soil acid phosphatase (mg/g/d); NP is soil neutral phosphatase (mg/g/d); ALP is soil alkaline (mg/g/d); SC is soil sucrase (mg/d/g); PPO is soil polyphenol oxidase (mg/g/2 h); CL is soil cellulase (mg/d/g); and β-XYS is soil β-xylosidase (μg/g/h).

Redundancy analysis was conducted between the soil physicochemical properties and the bacterial community and between the soil enzyme activity and the soil bacterial community (Figures 6A,B). The soil physicochemical properties and soil enzyme activity explained 93.38% of the variation in the soil bacterial community, and 71.73% of the variation could be explained by the first and second axes. The contribution rates of CEC, pH, TK and AK to the soil physicochemical properties were 15.1, 16.6, 16.3, and 13.2%, respectively, but these contributions were not significant (*p* < 0.05). CEC, pH and TK were positively correlated with *Gaiella* and negatively correlated with *Ellin6067*. Similarly, the contribution rates of CAT, NPT, SC and DHA to soil enzymes were 25.2, 18.4, 18.2, and 11.5%, respectively, but only the contributions of CAT and NPT were significant (*p* < 0.05). CAT and NPT were positively correlated with *Gaiella*, *Anaeromyxobacter*, and *Ilumatobacter*. The OY treatment was mainly distributed in the region of pH, TK, CEC, CAT and NPT, indicating that the soil bacterial diversity and richness changed greatly in the new rice-crab coculture mode paddy.

**Figure 7:**
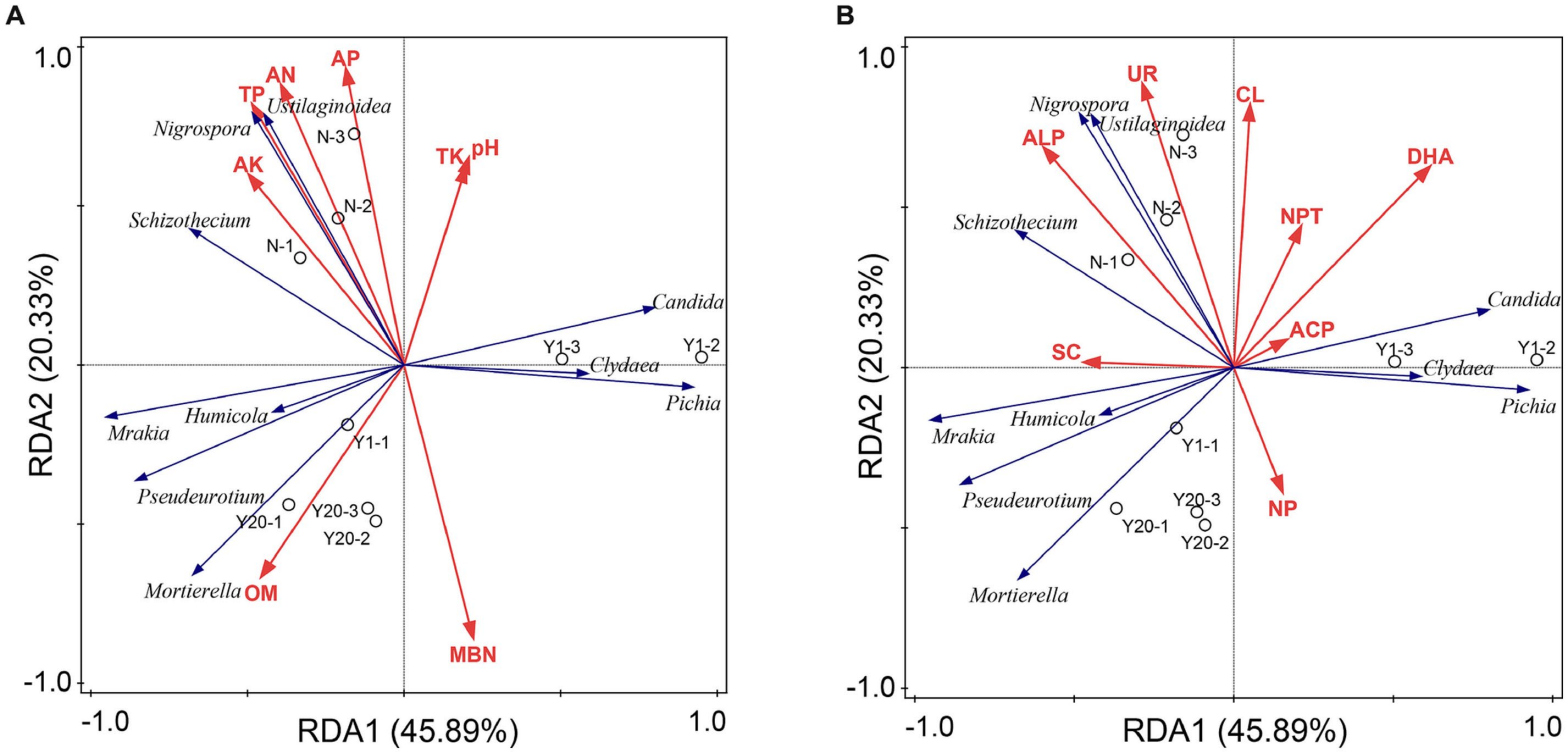
**(A)** Redundancy analysis of fungal communities and soil properties. TN is total nitrogen (g/kg); TP is total phosphorus (g/kg); TK is total potassium (g/kg); AN is available nitrogen (mg/kg); AP is available phosphorus (mg/kg); AK is available potassium (mg/kg); OM is organic matter (g/kg); CEC is cation exchange capacity (cmol/kg); MBC is microbial biomass carbon (mg/kg); MBN is microbial biomass nitrogen (mg/kg). **(B)** Redundancy analysis of fungal communities and soil enzymes. UR is soil urease (mg/d/g); NPT is soil protease (μg/g/2 h 50°C); DHA is soil dehydrogenase (μg/g/d); CAT is soil catalase (μmol/min); ACP is soil acid phosphatase (mg/g/d); NP is soil neutral phosphatase (mg/g/d); ALP is soil alkaline (mg/g/d); SC is soil sucrase (mg/d/g); PPO is soil polyphenol oxidase (mg/g/2 h); CL is soil cellulase (mg/d/g); and β-XYS is soil β-xylosidase (μg/g/h).

Redundancy analysis was also conducted between the soil physicochemical properties and soil enzyme activity and the soil fungal community (Figures 7A,B). The results showed that the soil physicochemical properties and soil enzyme activity explained 93.66% of the variation in the soil fungal community, among which the first and second axes explained 66.22% of the variation. The contribution rates of TP, OM, AN, and AK to the soil physicochemical properties were 24.6, 24.3, 11.9, and 11.0%, respectively, with TP and OM having a significant influence (*p* < 0.05). TP was significantly positively correlated with *Nigrospora*, *Schizothecium*, and *Ustilaginoidea*. OM was significantly positively correlated with *Mortierella*, *Pseudeurotium*, *Mrakia*, and *Humicola*. The contribution rates of ALP, DHA, SC and NPT to soil enzymes were 27.7, 27.3, 14.3, and 11.4%, respectively, with ALP and DHA having a significant effect (*p* < 0.05). ALP was significantly positively correlated with *Nigrospora*, *Schizothecium*, *Ustilaginoidea*, *Pseudeurotium*, *Mrakia*, and *Humicola*. DHA was significantly correlated with *Candida*, *Clydaea*, and *Pichia*. Th N treatment was mainly distributed in the region of TP, ALP and DHA, whereas the MY and OY treatments were in the region of OM, indicating that the change in OM content was the main factor affecting the soil fungal diversity and richness between the rice-crab coculture mode and the rice monoculture mode.

## 4 Discussion

We found that there were differences in the soil physicochemical properties, soil enzyme activity, and soil microbial community richness and diversity among the MY, OY and N treatments. As the duration increased, the richness and diversity of the microbial communities in the rice-crab coculture mode also changed (Figures 3A,B). The soil organic matter content and dehydrogenase activity were the main factors affecting the richness and diversity of the microbial communities (Figures 6A,B, 7A,B). The rice-crab coculture mode is different from the rice monoculture mode in terms of cultivation methods, especially in terms of nutrient inputs and the use of pesticides (Wang et al., 2014).

### 4.1 Effects of nutrient inputs on the richness and diversity of soil microbial communities

The richness and diversity of soil microorganisms are critical to soil quality, regulate nutrient uptake and resistance to adversity and are equally important for food security (Loeppmann et al., 2016). Exogenous nutrients such as organic and inorganic fertilizers have also been proven to indirectly affect the richness and diversity of microbial communities by changing the physicochemical properties of the soil, such as the soil porosity, pH, and organic carbon content (Siebert et al., 2018; Schmidt et al., 2019).

In our experiment, the duration of the rice-crab coculture increased the soil organic matter content, which had a significant effect on the richness and diversity of the soil microbial communities (Table 2 and Figure 7A). This finding was similar to the results of Su et al. (2014). Although no organic fertilizer was applied in the rice-crab coculture mode, the soil OM increased. We surmised that the increase in the soil organic matter content in this mode was due to crabs, which changed the type of nutrient input during rice growth. Soil physicochemical properties such as OM and pH are important factors that regulate the richness and diversity of the soil microbial community (Muhammad et al., 2021). We also found that the OM content was the main factor affecting soil microbial community. An increase in soil organic matter has been proven to improve soil physical structure and nutrient composition and provide resources for bacterial growth (Li et al., 2020). Iqbal et al. (2022) suggested that organic fertilizers could significantly change the composition of soil bacterial communities. Hu et al. (2022) reported that organic fertilizer could significantly increase the relative abundances of *α Proteus*, *acidic Proteus* and *delta Proteus* and decrease the relative abundance of *β Proteus*. However, the dominant bacterial species in N, OY and MY treatments, there were significant difference in *unidentified*, *uncultured*, *uncultured_bacterium* and *Thiobacillus*. Among them, there was a significant difference in the relative abundance of *uncultured* between N and MY treatments, and *uncultured_bacterium* between N and OY treatments. This might be because in the production process of the rice-crab coculture mode, no pesticides are used, and the soil organic matter content increases (Frac et al., 2021; Hugoni et al., 2021). This indicated that soil bacterial communities in the rice-crab coculture mode still need research. According to our results, the soil fungal community changed more significantly than did the bacterial community among the treatments. This finding was similar to the results of Xiao et al. (2022) and Wang et al. (2014). These authors demonstrated that optimizing tillage methods can effectively increase the soil organic matter content and cause changes in soil fungal communities. We also found that the OM content differed among the MY, OY and N treatments. An increase in soil organic matter directly caused an increase in the soluble organic carbon content, which regulated the diversity and richness of soil fungal communities (Hamman et al., 2007; Melero et al., 2006). Lee et al. (2013) conducted experiments involving the application of organic fertilizer, chemical fertilizer and no fertilizer in cabbage fields and reported that the application of organic fertilizer was the main reason for the differentiation of soil fungal communities through an increase in the soil organic matter content. Liu et al. (2019) concluded that soil organic carbon was the main reason for the changes in the Shannon–Wiener index and richness index of soil fungal communities in paddy fields through 30-year field experiments with organic and inorganic fertilizers.

### 4.2 Effects of differences in pesticide use on the richness and diversity of soil microbial communities

Pesticides, similar to fertilizers, improve the ability of crops to resist adverse natural environmental conditions and improve agricultural production. In this study, in addition to the use of exogenous crabs in the rice-crab coculture mode, the lack of pesticides and herbicides was also the main reason for the change in the microbial community (Wolejko et al., 2020; Micuti et al., 2020).

Soil enzymes primarily originate from the secretions of animals, plant roots, and microbial cells within the soil. Enzyme activities can serve as indicators of soil microbial diversity and abundance. The results of this experiment showed significant differences in the activities of UR, DHA, ALP and CL in the soils from the Y20 and N treatments (Table 3). Among them, the explanations of DHA and ALP for the differences in the microbial communities reached a significant level in the redundancy analysis. DHA is an enzyme that exists in microbial cells, is related to the microbial respiration process, and is used as an indicator to evaluate overall microbial activity in soil (Riah et al., 2014). Stepniewska et al. (2007) suggested that fonofos pesticide could significantly reduce the activity of soil dehydrogenase, and the inhibitory effect intensified with increasing drug dose. Filimon et al. (2021) drew a similar conclusion, suggesting that the use of fluoxifen could rapidly reduce the activity of soil dehydrogenase, phosphatase, urease and protease and have an impact on the microbial flora. UR, ALP and CL are enzymes related to the soil nitrogen cycle, phosphorus cycle and carbon cycle, respectively, and their final metabolites can be used as energy sources by soil microorganisms (Deng and Tabatabai, 1994). Compared with the rice-crab coculture mode, the rice monoculture mode does not need to consider the survival of crabs, so herbicides and pesticides are used during rice growth to obtain the maximum yield. Since microorganisms are sensitive to changes in the external environment, pesticide intervention disrupts the soil primary balance and directly changes the structure and function of the microbial community (Widenfalk et al., 2008). When pesticides enter the soil, microbes overproduce enzymes that breakdown toxic compounds, such as those present in pesticides and herbicides, resulting in the acquisition of new genes within microbial communities and the alteration of microbial community structure and function (Rangasamy et al., 2018). Thus, we presumed that the difference in soil biodiversity between the coculture and monoculture modes was caused by pesticide intervention. Moreover, the difference in biodiversity between MY and OY was caused by the duration of pesticides discontinuation (Walder et al., 2022). They reported that pesticides have potential long-term effects on soil function and can alter the soil microbial community and that the effects on fungal diversity and richness are different from those on bacterial diversity and richness.

In this experiment, although the MY, OY and N treatments differed greatly in terms of cultivation methods and duration, and the soil samples collected were not from the same paddy field, which may have had an impact on the results of this experiment. Nevertheless, the differences in the test results of the different treatments may reflect the change trends in soil nutrients, enzyme activity and microorganisms under different cultivation systems. There is a need to explore soil nutrient and energy cycling in paddy fields with different cultivation systems.

## 5 Conclusion

The rice-crab coculture mode is a comprehensive agricultural system that integrates rice and crab cultivation and can change soil physicochemical properties, enzyme activity and soil biodiversity. Among these changes, the changes in the soil organic matter content and dehydrogenase activity are most closely related to the changes in the soil microbial community. In the rice-crab coculture mode, the richness and diversity of microbial communities continuously change, leading to differences in the dominant flora between long-term coculturing and newly-established coculturing paddies. Furthermore, the long-term coculture mode has a positive effect on increasing soil organic matter and sequestering soil carbon, which holds potential for green and sustainable agricultural development.

## Data Availability

The original contributions presented in the study are included in the article/supplementary material, further inquiries can be directed to the corresponding author.
